# A tetra­nuclear cubane-like nickel(II) complex with a tridentate salicyl­idene­imine Schiff base ligand: tetra­kis­[μ_3_-4-methyl-*N*-(2-oxidophen­yl)salicylideneiminato]tetra­kis­[methano­lnickel(II)] methanol 0.8-solvate

**DOI:** 10.1107/S2056989016017722

**Published:** 2016-11-10

**Authors:** Gordana Pavlović, Mihael Majer, Marina Cindrić

**Affiliations:** a University of Zagreb, Faculty of Textile Technology, Laboratory of Applied Chemistry, Prilaz baruna Filipovića 28a, HR-10000 Zagreb, Croatia; b University of Zagreb, Faculty of Science, Department of Chemistry, Horvatovac 102a, HR-10000 Zagreb, Croatia

**Keywords:** crystal structure, nickel cubane-like tetra­mers, Schiff bases

## Abstract

The tetra­nuclear title complex, [Ni_4_(C_14_H_11_NO_2_)_4_(CH_3_OH)_4_]·0.8CH_3_OH, has a distorted cubane topology shaped by four Schiff base ligands. The cubane [Ni_4_(μ_3_-O_4_)] core is formed *via* the O atoms from the Schiff base ligands. The octa­hedrally coordinated Ni^II^ ions occupy alternating vertices of the cube. Each Ni^II^ ion is coordinated by one *O*,*N*,*O*′-tridentate dianionic ligand, two O atoms of oxidophenyl groups from adjacent ligands and the O atom of a coordinating methanol mol­ecule. The cubane core is stabilized *via* an intra­molecular O—H⋯O hydrogen bond between the hy­droxy group of the coordinating methanol mol­ecules and the phenolate O atom of the aldehyde Schiff base fragment. Additional stabilization is obtained *via* intra­molecular C—H⋯O hydrogen bonds involving aromatic C—H groups and the oxygen atoms of adjacent methanol mol­ecules. In the crystal, complex mol­ecules are linked into chains parallel to the *c* axis *via* weak C—H⋯O hydrogen bonds. The partial-occupancy disordered methanol solvent mol­ecule has a site occupancy of 0.8 and is linked to the tetra­nuclear unit *via* an inter­molecular C—H⋯O hydrogen bond involving a phenolate O atom.

## Abstract 2

In the continuation of our research on Ni_4_ *L*_4_ cubane-like clusters with salicyl­idene­imine Schiff base type of ligands, the crystal and mol­ecular structure of an analogous complex of formula [Ni_4_ *L*_4_(CH_3_OH)_4_]·0.8CH_3_OH [*L* = *N*-(2-hy­droxy-4-methyl­phen­yl)salicyl­idene­imine] is reported in order to investigate the influence of the methyl-group position of the ligand on the geometry of the cubane core, as well as on the supra­molecular assembling of the cluster units and consequently on the magnetostructural properties of this class of compounds.

## Chemical context   

Octa­hedrally coordinated Ni^II^ atoms are paramagnetic and spanned by an appropriate bridging ligand. They can be organized into polynuclear units of different nuclearity with potential practical applications as nanomagnetic devices, switches and sensors or single-mol­ecule magnets (Ji *et al.*, 2009 ▸; Karmakar & Khanra, 2014 ▸; Kou *et al.*, 2010 ▸; Osa *et al.*, 2004 ▸; Perlepe *et al.*, 2014 ▸; Pardo *et al.*, 2008 ▸; Papatrianta­fyll­op­oulou *et al.*, 2008 ▸; Polyakov *et al.*, 2012 ▸). One of the major requirements in designing single-mol­ecule magnets (SMM) is to obtain slight structural changes in enduring metal–organic frameworks. The important subject in this field is the relationship between the magnetic behaviour of the mol­ecule and its microenvironment. It is known that any symmetry decrease manifested as reduced symmetry of the arrangement of ligands around metal atoms (no imposed crystallographic symmetry within complex mol­ecule), crystallographic disorders of terminal groups of the ligand mol­ecules, existence of two or more crystallographically independent complex mol­ecules in one asymmetric unit or weakly inter­acting solvent mol­ecules (Lawrence *et al.*, 2008 ▸; Cotton *et al.*, 2007 ▸) influences the magnetic properties strongly. Although it has been shown that Ni_4_O_4_ cubane-like Ni units have a rather robust structure with persistent geometrical parameters, even weak inter­actions influence their magnetic behaviour, causing almost indiscernible distortions of the cubane core. The particular importance of the Ni—μ_3_-O—Ni bond angles is emphasized in the modelling of the intra­molecular magnetic inter­actions. Previous investigations showed that ferromagnetic inter­actions are associated with angles close to 90° and anti­ferromagnetic inter­actions with larger angles (Ballester *et al.*, 1992 ▸; Bertrand *et al.*, 1978 ▸; Gladfelter *et al.*, 1981 ▸; Halcrow *et al.*, 1995 ▸; Petit *et al.*, 2012 ▸; Zhang *et al.*, 2012 ▸). Therefore, the cubane Ni_4_ *L*_4_ topology represents a plethora of possibilities in the design of single-mol­ecule magnets.
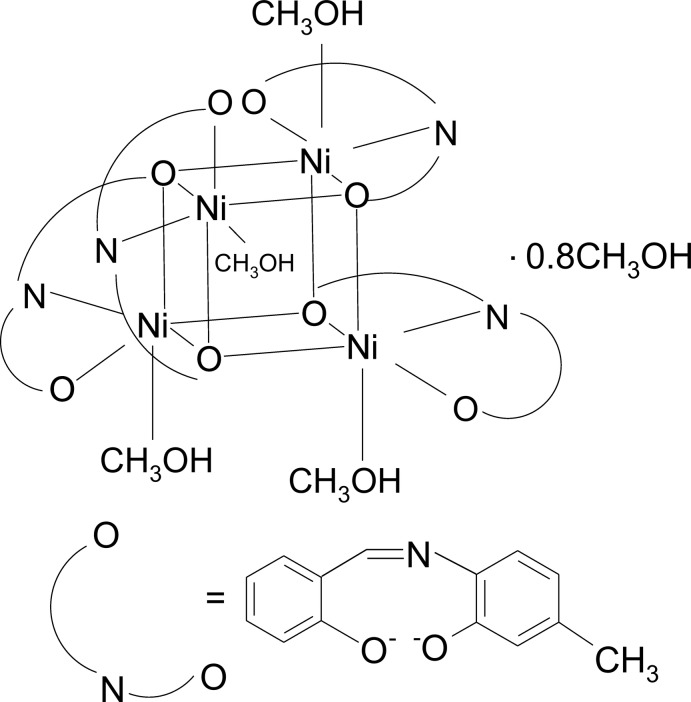


## Structural commentary   

**Figure 1:**
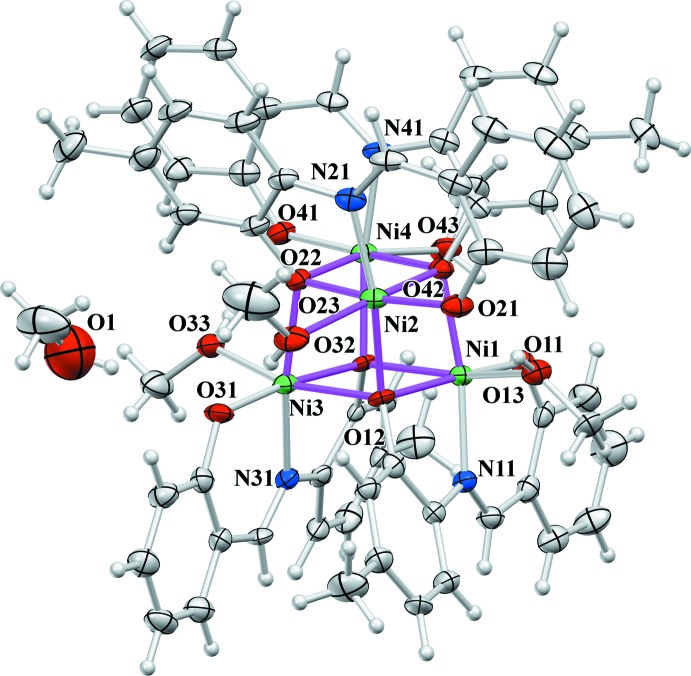
The mol­ecular structure of the title compound, with displacement ellipsoids drawn at the 50% probability level. The edges of the Ni_4_O_4_ cubane are denoted in violet.

In the title compound, each Ni^II^ ion (Fig. 1 ▸) is six-coordinated by one phenolate oxygen atom [1.957 (3)–1.975 (3) Å], one imino nitro­gen atom [1.967 (4)–1.976 (4) Å] and the oxygen atom of the N-substituent moiety [2.043 (3)–2.083 (3) Å] from a dianionic tridentate Schiff base ligand as well as by the μ_3_-O oxygen atom of the N-substituent moiety of another ligand mol­ecule. The sixth coordination site *trans* to the μ_3_-O oxygen is provided by the oxygen atom from a neutral MeOH monodentate ligand [2.071 (4)-2.137 (4) Å]. Two oxygen atoms and one nitro­gen atom from the same salicylaldiminato moiety form two five- and six-membered chelate rings fused across the Ni—N bond. The trend of values of the Ni—O bond lengths is Ni—O(phenolate) < Ni—O(CH_3_OH) < Ni—μ_3_-O. The bond angles indicate that nickel(II) ions exhibit a distorted octa­hedral environment with the *X*—Ni—*X* (*X* = O, N) angles in the ranges 77.90 (12)–101.58 (13)° and 163.76 (13)–172.48 (13)° for *cis* and *trans* angles, respectively. The deformation from the ideal tetra­hedral geometry around the μ_3_-O oxygen atoms is also suggested by the values of the Ni—μ_3_-O—Ni angles which fall in the range 91.58 (12)–102.38 (13)°. The significant double-bond character of the C—N bonds [1.284 (6)–1.285 (6) Å] clearly indicates the presence of the imino tautomeric form of all four Schiff base ligands. The C*sp* ^2^—N single bonds are in the range 1.413 (6)–1.427 (6) Å.

## Supra­molecular features   

**Figure 2:**
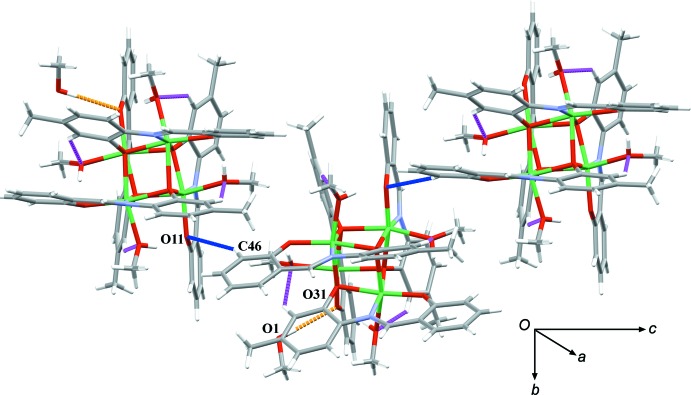
The supra­molecular assembly of the complex units of the title compound *via* intra- and inter­molecular hydrogen bonds. The hydrogen bonds are denoted as follows: intra­molecular in magenta, inter­molecular with the methanol solvent mol­ecule in orange and inter­molecular linking cluster units in blue.

The coordinating methanol mol­ecules participate in the formation of intra­molecular hydrogen bonds with the phenolate O atoms of the Schiff base ligand (O11, O21, O31 and O41). These intra­molecular hydrogen bonds (Table 1 ▸, Fig. 2 ▸) span across four of six cubane faces influencing the values of the Ni⋯Ni separations [3.081 (1) –3.323  (1) Å]. The methanol mol­ecule of crystallization inter­acts with the complex units *via* an inter­molecular hydrogen bond with the phenolate O31 atom. In the crystal, the Ni_4_ *L*_4_ complex mol­ecules are linked into chains running parallel to the *c* axis by weak C—H⋯O hydrogen bonds between the C46 aromatic carbon atom and the O11 phenolate oxygen atom (Table 1 ▸). In the framework of our research on this type of Ni_4_ *L*_4_ units, we have published analogous Ni_4_ *L*_4_ cubane-like units with the *N*-(2-hy­droxy-5-methyl­phen­yl)salicyl­idene­imine ligand (Cindrić *et al.*, 2016 ▸). In these compounds, similar C—H⋯O hydrogen bonds involving an aromatic C—H group and one phenolate oxygen atom result in the formation of discrete centrosymmetric dimers.

## Synthesis and crystallization   

The title compound was prepared by mixing a methano­lic solution of Ni(O_2_CMe)_2_·4H_2_O (1 mmol in 10 ml) and a methano­lic solution of the Schiff base ligand *N*-(2-hy­droxy-4-methyl­phen­yl)salicyl­idene­imine (1 mmol in 10 ml) at room temperature. After two days, green prismatic single crystals suitable for X-ray analysis were obtained on slow evaporation of the solvent. Yield 58%: Analysis calculated (without lattice solvent) (%) for C_60_H_60_N_4_Ni_4_O_12_: C, 57.02; H, 4.78; N, 4.43; Ni, 18.57. Found: C,56.70; H, 4.80; N, 4.29; Ni, 18.50. Spectroscopic analysis, IR (ATR, cm^−1^): 3406 (*b*,*m*), 3056 (*m*), 3007 (*m*), 2917 (*m*), 2793 (*m*), 1604 (*vs*), 1531 (*s*), 1490 (*vs*), 1378 (*m*), 1305 (*s*), 1226 (*s*), 1127 (*s*), 1034 (*m*), 825 (*s*), 750 (*s*), 522 (*m*).

## Refinement   

Crystal data, data collection and structure refinement details are summarized in Table 2 ▸. The methanol molecule is disordered and was refined with a site-occupancy factor of 0.80. The C-bound hydrogen atoms were placed in geometrically idealized positions and constrained to ride on their parent atoms, with C—H = 0.93–0.96 Å, and with *U*_iso_(H) = 1.2*U*_eq_(C) or 1.5*U*_eq_(C) for methyl H atoms. A rotating model was used for the methyl groups. The hy­droxy H atoms of the coordinating methanol mol­ecules were firstly found in a difference Fourier map and then refined by constraining the C—H bond length to be 0.84 (2) Å and the isotropic displacement parameters to be 1.2 times the equivalent isotropic displacement parameters of the parent oxygen atoms. The hy­droxy H atom of the disordered methanol mol­ecule was located in a difference Fourier map and refined with fixed coordinates and *U*_iso_(H) = 1.5*U*_eq_(O). Displacement restraints (SIMU and DELU; Sheldrick, 2015 ▸) were applied to the disordered partial methanol mol­ecule.

## Supplementary Material

Crystal structure: contains datablock(s) I, global. DOI: 10.1107/S2056989016017722/rz5196sup1.cif

Structure factors: contains datablock(s) I. DOI: 10.1107/S2056989016017722/rz5196Isup2.hkl

CCDC reference: 1515300

Additional supporting information: crystallographic information; 3D view; checkCIF report

## Figures and Tables

**Table 1:** Hydrogen-bond geometry (Å, °)

*D*—H⋯*A*	*D*—H	H⋯*A*	*D*⋯*A*	*D*—H⋯*A*
O13—H13*O*⋯O21	0.84 (2)	1.89 (3)	2.688 (5)	161 (6)
O23—H23*O*⋯O31	0.84 (2)	1.87 (2)	2.709 (5)	177 (7)
O33—H33*O*⋯O41	0.82 (2)	1.88 (3)	2.645 (5)	155 (7)
O43—H43*O*⋯O11	0.81 (2)	1.93 (3)	2.686 (5)	155 (7)
C46—H46⋯O11^i^	0.93	2.55	3.307 (6)	139
C110—H110⋯O23	0.93	2.42	3.177 (6)	138
C210—H210⋯O33	0.93	2.44	3.175 (6)	136
C310—H310⋯O43	0.93	2.48	3.218 (6)	137
C410—H410⋯O13	0.93	2.45	3.189 (6)	136
O1—H11*O*⋯O31	0.83	2.2100	3.034 (8)	177

Symmetry code: (i) 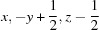.

**Table 2:** Experimental details

Crystal data	
Chemical formula	[Ni_4_(C_14_H_11_NO_2_)_4_(CH_4_O)_4_]·0.8CH_4_O
*M*_r_	1289.59
Crystal system, space group	Monoclinic, *P*2_1_/*c*
Temperature (K)	296
*a*, *b*, *c* (Å)	22.5810 (5), 13.7701 (3), 18.5242 (4)
β (°)	92.125 (2)
*V* (Å^3^)	5756.0 (2)
*Z*	4
Radiation type	Mo *K*α
μ (mm^−1^)	1.36
Crystal size (mm)	0.18 × 0.11 × 0.09

Data collection	
Diffractometer	Oxford Diffraction Xcalibur Sapphire3
Absorption correction	Multi-scan (*CrysAlis PRO*; Oxford Diffraction, 2010 ▸)
*T*_min_, *T*_max_	0.928, 1.000
No. of measured, independent and observed [*I* > 2σ(*I*)] reflections	23546, 12324, 6994
*R*_int_	0.069
(sin θ/λ)_max_ (Å^−1^)	0.639

Refinement	
*R*[*F* ^2^ > 2σ(*F* ^2^)], *wR*(*F* ^2^), *S*	0.068, 0.130, 0.99
No. of reflections	12324
No. of parameters	760
No. of restraints	11
H-atom treatment	H atoms treated by a mixture of independent and constrained refinement
Δρ_max_, Δρ_min_ (e Å^−3^)	0.94, −0.52

Computer programs: *CrysAlis PRO* (Oxford Diffraction, 2010 ▸), *SHELXS2014* (Sheldrick, 2008 ▸), *SHELXL2014* (Sheldrick, 2015 ▸) and *Mercury* (Macrae *et al.*, 2006 ▸).

## supplementary crystallographic information

### Crystal data

**Table:**

[Ni_4_(C_14_H_11_NO_2_)_4_(CH_4_O)_4_]·0.8CH_4_O	*D*_x_ = 1.488 Mg m^−^^3^
*M**_r_* = 1289.59	Melting point: 651 K
Monoclinic, *P*2_1_/*c*	Mo *K*α radiation, λ = 0.71073 Å
*a* = 22.5810 (5) Å	Cell parameters from 4839 reflections
*b* = 13.7701 (3) Å	θ = 4.1–29.0°
*c* = 18.5242 (4) Å	µ = 1.36 mm^−^^1^
β = 92.125 (2)°	*T* = 296 K
*V* = 5756.0 (2) Å^3^	Prism, green
*Z* = 4	0.18 × 0.11 × 0.09 mm
*F*(000) = 2682	

### Data collection

**Table:**

Oxford Diffraction Xcalibur Sapphire3 diffractometer	6994 reflections with *I* > 2σ(*I*)
Radiation source: Enhance (Mo) X-ray Source	*R*_int_ = 0.069
ω scans	θ_max_ = 27.0°, θ_min_ = 4.1°
Absorption correction: multi-scan (CrysAlis PRO; Oxford Diffraction, 2010)	*h* = −11→28
*T*_min_ = 0.928, *T*_max_ = 1.000	*k* = −15→17
23546 measured reflections	*l* = −23→23
12324 independent reflections	500 standard reflections every 90 min

### Refinement

**Table:**

Refinement on *F*^2^	11 restraints
Least-squares matrix: full	Hydrogen site location: mixed
*R*[*F*^2^ > 2σ(*F*^2^)] = 0.068	H atoms treated by a mixture of independent and constrained refinement
*wR*(*F*^2^) = 0.130	*w* = 1/[σ^2^(*F*_o_^2^) + (0.0392*P*)^2^] where *P* = (*F*_o_^2^ + 2*F*_c_^2^)/3
*S* = 0.99	(Δ/σ)_max_ < 0.001
12324 reflections	Δρ_max_ = 0.94 e Å^−^^3^
760 parameters	Δρ_min_ = −0.52 e Å^−^^3^

### Special details

**Table:**

Geometry. All esds (except the esd in the dihedral angle between two l.s. planes) are estimated using the full covariance matrix. The cell esds are taken into account individually in the estimation of esds in distances, angles and torsion angles; correlations between esds in cell parameters are only used when they are defined by crystal symmetry. An approximate (isotropic) treatment of cell esds is used for estimating esds involving l.s. planes.

### Fractional atomic coordinates and isotropic or equivalent isotropic displacement parameters (Å^2^)

**Table:**

	*x*	*y*	*z*	*U*_iso_*/*U*_eq_	Occ. (<1)
Ni1	0.22977 (3)	0.33062 (4)	0.22684 (3)	0.01959 (16)	
Ni2	0.28942 (3)	0.53003 (4)	0.20271 (4)	0.02273 (17)	
Ni3	0.19370 (3)	0.47067 (4)	0.08836 (3)	0.02057 (16)	
Ni4	0.30539 (3)	0.34270 (4)	0.09204 (3)	0.02114 (17)	
N11	0.14516 (18)	0.3221 (3)	0.2498 (2)	0.0212 (10)	
N21	0.36757 (19)	0.5812 (3)	0.1782 (2)	0.0243 (10)	
N31	0.11291 (18)	0.4170 (3)	0.0853 (2)	0.0211 (10)	
N41	0.39195 (18)	0.3621 (3)	0.0962 (2)	0.0233 (10)	
O11	0.24166 (15)	0.1885 (2)	0.22879 (18)	0.0241 (8)	
O13	0.25424 (16)	0.3688 (2)	0.33300 (19)	0.0275 (9)	
O21	0.30451 (15)	0.5418 (2)	0.30751 (18)	0.0274 (9)	
O22	0.28278 (14)	0.4994 (2)	0.09477 (18)	0.0224 (8)	
O23	0.24994 (16)	0.6682 (2)	0.1800 (2)	0.0322 (9)	
O31	0.16684 (15)	0.6065 (2)	0.08120 (19)	0.0275 (9)	
O32	0.21570 (14)	0.3282 (2)	0.10809 (16)	0.0185 (7)	
O33	0.20342 (16)	0.4437 (2)	−0.02057 (19)	0.0273 (9)	
O41	0.30032 (15)	0.3357 (2)	−0.01353 (17)	0.0257 (8)	
O42	0.31442 (14)	0.3721 (2)	0.20030 (17)	0.0207 (8)	
O43	0.31349 (17)	0.1940 (2)	0.1165 (2)	0.0309 (9)	
O12	0.20613 (14)	0.4745 (2)	0.20637 (17)	0.0195 (8)	
C11	0.1986 (2)	0.1234 (3)	0.2342 (3)	0.0236 (12)	
C12	0.1378 (2)	0.1468 (3)	0.2425 (3)	0.0238 (12)	
C13	0.0963 (3)	0.0704 (4)	0.2466 (3)	0.0299 (13)	
H13	0.0566	0.0858	0.2515	0.036*	
C14	0.1118 (3)	−0.0238 (4)	0.2436 (3)	0.0371 (15)	
H14	0.0834	−0.0724	0.2461	0.045*	
C15	0.1714 (3)	−0.0472 (4)	0.2369 (3)	0.0444 (17)	
H15	0.1830	−0.1119	0.2356	0.053*	
C16	0.2131 (3)	0.0253 (4)	0.2321 (3)	0.0341 (14)	
H16	0.2525	0.0079	0.2273	0.041*	
C17	0.1147 (2)	0.2434 (3)	0.2504 (3)	0.0250 (12)	
H17	0.0741	0.2493	0.2565	0.030*	
C18	0.1214 (2)	0.4156 (3)	0.2649 (3)	0.0222 (12)	
C19	0.1570 (2)	0.4941 (3)	0.2444 (3)	0.0234 (12)	
C21	0.3566 (2)	0.5616 (4)	0.3385 (3)	0.0294 (13)	
C22	0.4074 (2)	0.5907 (3)	0.3019 (3)	0.0284 (13)	
C23	0.4604 (3)	0.6102 (4)	0.3420 (3)	0.0374 (15)	
H23	0.4938	0.6291	0.3176	0.045*	
C24	0.4641 (3)	0.6022 (4)	0.4154 (4)	0.0434 (16)	
H24	0.4997	0.6147	0.4406	0.052*	
C25	0.4146 (3)	0.5752 (4)	0.4517 (3)	0.0402 (16)	
H25	0.4167	0.5701	0.5018	0.048*	
C26	0.3621 (3)	0.5558 (4)	0.4143 (3)	0.0365 (15)	
H26	0.3291	0.5382	0.4400	0.044*	
C27	0.4100 (2)	0.6017 (3)	0.2238 (3)	0.0300 (14)	
H27	0.4450	0.6255	0.2056	0.036*	
C28	0.3690 (2)	0.5989 (3)	0.1032 (3)	0.0240 (12)	
C29	0.3212 (2)	0.5602 (3)	0.0612 (3)	0.0235 (12)	
C31	0.1121 (2)	0.6349 (4)	0.0894 (3)	0.0249 (12)	
C32	0.0624 (2)	0.5713 (4)	0.0934 (3)	0.0240 (12)	
C33	0.0055 (2)	0.6113 (4)	0.1023 (3)	0.0310 (13)	
H33	−0.0267	0.5696	0.1052	0.037*	
C34	−0.0039 (3)	0.7102 (4)	0.1070 (3)	0.0334 (14)	
H34	−0.0417	0.7350	0.1128	0.040*	
C35	0.0446 (3)	0.7713 (4)	0.1028 (3)	0.0338 (14)	
H35	0.0391	0.8382	0.1057	0.041*	
C36	0.1001 (2)	0.7353 (3)	0.0947 (3)	0.0299 (13)	
H36	0.1315	0.7787	0.0924	0.036*	
C37	0.0650 (2)	0.4667 (4)	0.0890 (3)	0.0249 (12)	
H37	0.0294	0.4329	0.0889	0.030*	
C38	0.1137 (2)	0.3143 (3)	0.0758 (3)	0.0206 (11)	
C39	0.1696 (2)	0.2716 (3)	0.0836 (2)	0.0171 (11)	
C41	0.3423 (2)	0.3634 (3)	−0.0560 (3)	0.0262 (12)	
C42	0.4017 (2)	0.3897 (3)	−0.0316 (3)	0.0252 (12)	
C43	0.4411 (2)	0.4243 (3)	−0.0826 (3)	0.0290 (13)	
H43	0.4788	0.4435	−0.0664	0.035*	
C44	0.4271 (3)	0.4312 (4)	−0.1544 (3)	0.0342 (14)	
H44	0.4543	0.4545	−0.1867	0.041*	
C45	0.3702 (3)	0.4022 (4)	−0.1782 (3)	0.0344 (14)	
H45	0.3598	0.4054	−0.2272	0.041*	
C46	0.3298 (2)	0.3693 (3)	−0.1312 (3)	0.0292 (13)	
H46	0.2927	0.3501	−0.1493	0.035*	
C47	0.4230 (2)	0.3847 (3)	0.0420 (3)	0.0248 (12)	
H47	0.4628	0.3990	0.0514	0.030*	
C48	0.4152 (2)	0.3554 (3)	0.1684 (3)	0.0242 (12)	
C49	0.3717 (2)	0.3562 (3)	0.2224 (3)	0.0211 (12)	
C110	0.1400 (2)	0.5863 (3)	0.2631 (3)	0.0236 (12)	
H110	0.1637	0.6384	0.2507	0.028*	
C111	0.0882 (2)	0.6046 (4)	0.3001 (3)	0.0297 (13)	
C112	0.0537 (2)	0.5253 (4)	0.3188 (3)	0.0305 (13)	
H112	0.0187	0.5354	0.3428	0.037*	
C113	0.0707 (2)	0.4323 (4)	0.3022 (3)	0.0281 (13)	
H113	0.0477	0.3800	0.3163	0.034*	
C114	0.0694 (3)	0.7072 (4)	0.3181 (3)	0.0442 (17)	
H11A	0.0475	0.7345	0.2775	0.066*	
H11B	0.1038	0.7461	0.3289	0.066*	
H11C	0.0447	0.7060	0.3592	0.066*	
C115	0.2231 (3)	0.3516 (4)	0.3974 (3)	0.0368 (15)	
H11D	0.1966	0.4047	0.4055	0.055*	
H11E	0.2510	0.3462	0.4376	0.055*	
H11F	0.2009	0.2924	0.3925	0.055*	
C210	0.3156 (2)	0.5828 (3)	−0.0120 (3)	0.0269 (13)	
H210	0.2835	0.5587	−0.0394	0.032*	
C211	0.3570 (3)	0.6408 (4)	−0.0447 (3)	0.0343 (15)	
C212	0.4047 (3)	0.6756 (4)	−0.0026 (3)	0.0350 (15)	
H212	0.4333	0.7131	−0.0242	0.042*	
C213	0.4104 (2)	0.6562 (3)	0.0697 (3)	0.0327 (14)	
H213	0.4423	0.6817	0.0966	0.039*	
C214	0.3498 (3)	0.6647 (4)	−0.1238 (3)	0.0439 (16)	
H21A	0.3089	0.6777	−0.1357	0.066*	
H21B	0.3629	0.6108	−0.1518	0.066*	
H21C	0.3731	0.7210	−0.1342	0.066*	
C215	0.2791 (3)	0.7600 (5)	0.1679 (4)	0.076 (3)	
H21D	0.3086	0.7711	0.2056	0.115*	
H21E	0.2504	0.8114	0.1679	0.115*	
H21F	0.2977	0.7584	0.1221	0.115*	
C310	0.1757 (2)	0.1729 (3)	0.0683 (2)	0.0209 (11)	
H310	0.2132	0.1448	0.0715	0.025*	
C311	0.1274 (2)	0.1162 (3)	0.0485 (3)	0.0254 (12)	
C312	0.0722 (3)	0.1590 (4)	0.0433 (3)	0.0325 (14)	
H312	0.0392	0.1213	0.0309	0.039*	
C313	0.0651 (2)	0.2569 (4)	0.0562 (3)	0.0280 (13)	
H313	0.0276	0.2846	0.0518	0.034*	
C314	0.1350 (3)	0.0092 (3)	0.0349 (3)	0.0392 (15)	
H31A	0.1158	−0.0077	−0.0104	0.059*	
H31B	0.1176	−0.0270	0.0730	0.059*	
H31C	0.1765	−0.0058	0.0335	0.059*	
C315	0.1592 (2)	0.4417 (4)	−0.0765 (3)	0.0440 (16)	
H31D	0.1351	0.3848	−0.0716	0.066*	
H31E	0.1775	0.4404	−0.1225	0.066*	
H31F	0.1348	0.4986	−0.0736	0.066*	
C410	0.3899 (2)	0.3465 (3)	0.2940 (3)	0.0223 (12)	
H410	0.3617	0.3438	0.3293	0.027*	
C411	0.4501 (2)	0.3407 (3)	0.3147 (3)	0.0255 (12)	
C412	0.4914 (2)	0.3417 (3)	0.2607 (3)	0.0316 (13)	
H412	0.5316	0.3385	0.2737	0.038*	
C413	0.4745 (2)	0.3474 (3)	0.1891 (3)	0.0299 (13)	
H413	0.5030	0.3458	0.1542	0.036*	
C414	0.4682 (2)	0.3359 (4)	0.3931 (3)	0.0337 (14)	
H41A	0.4592	0.2727	0.4116	0.051*	
H41B	0.4470	0.3842	0.4192	0.051*	
H41C	0.5100	0.3476	0.3989	0.051*	
C415	0.3597 (3)	0.1311 (4)	0.1015 (4)	0.059 (2)	
H41D	0.3731	0.1439	0.0539	0.088*	
H41E	0.3460	0.0652	0.1041	0.088*	
H41F	0.3917	0.1407	0.1363	0.088*	
H13O	0.267 (3)	0.426 (2)	0.335 (3)	0.071*	
H23O	0.224 (2)	0.648 (4)	0.151 (3)	0.071*	
H33O	0.227 (2)	0.399 (3)	−0.026 (4)	0.071*	
H43O	0.299 (3)	0.180 (4)	0.154 (2)	0.071*	
O1	0.1877 (4)	0.6934 (5)	−0.0662 (4)	0.114 (3)	0.8
H11O	0.1835	0.6704	−0.0255	0.137*	0.8
C1	0.2122 (4)	0.7851 (6)	−0.0592 (6)	0.079 (3)	0.8
H1A	0.1812	0.8320	−0.0537	0.118*	0.8
H1B	0.2336	0.8002	−0.1016	0.118*	0.8
H1C	0.2387	0.7869	−0.0176	0.118*	0.8

### Atomic displacement parameters (Å^2^)

**Table:**

	*U*^11^	*U*^22^	*U*^33^	*U*^12^	*U*^13^	*U*^23^
Ni1	0.0153 (3)	0.0220 (3)	0.0217 (4)	0.0002 (3)	0.0027 (3)	0.0003 (3)
Ni2	0.0162 (4)	0.0247 (3)	0.0274 (4)	−0.0023 (3)	0.0025 (3)	−0.0023 (3)
Ni3	0.0146 (3)	0.0231 (3)	0.0242 (4)	−0.0018 (3)	0.0024 (3)	0.0011 (3)
Ni4	0.0146 (3)	0.0256 (3)	0.0234 (4)	−0.0013 (3)	0.0029 (3)	−0.0025 (3)
N11	0.018 (2)	0.025 (2)	0.021 (2)	0.000 (2)	0.0033 (19)	0.0007 (18)
N21	0.020 (2)	0.021 (2)	0.032 (3)	−0.002 (2)	0.001 (2)	−0.0069 (19)
N31	0.020 (2)	0.022 (2)	0.021 (2)	−0.003 (2)	0.004 (2)	0.0027 (18)
N41	0.015 (2)	0.026 (2)	0.029 (3)	0.0000 (19)	0.006 (2)	−0.0075 (19)
O11	0.020 (2)	0.0251 (18)	0.027 (2)	0.0013 (16)	0.0009 (17)	0.0021 (15)
O13	0.025 (2)	0.0307 (19)	0.027 (2)	−0.0031 (18)	0.0051 (18)	−0.0027 (17)
O21	0.019 (2)	0.033 (2)	0.029 (2)	−0.0055 (17)	0.0020 (17)	−0.0085 (16)
O22	0.0179 (19)	0.0233 (18)	0.026 (2)	−0.0032 (15)	0.0043 (16)	0.0035 (15)
O23	0.025 (2)	0.0209 (19)	0.052 (3)	−0.0084 (18)	0.0059 (19)	−0.0012 (18)
O31	0.0141 (19)	0.0273 (19)	0.041 (2)	0.0027 (17)	0.0059 (18)	0.0044 (16)
O32	0.0133 (18)	0.0243 (17)	0.0186 (18)	−0.0042 (16)	0.0079 (15)	−0.0012 (15)
O33	0.020 (2)	0.040 (2)	0.022 (2)	−0.0008 (17)	0.0019 (17)	−0.0006 (17)
O41	0.022 (2)	0.0330 (19)	0.023 (2)	−0.0055 (17)	0.0057 (17)	−0.0035 (16)
O42	0.0150 (19)	0.0256 (18)	0.0214 (19)	−0.0016 (16)	0.0002 (16)	−0.0005 (15)
O43	0.027 (2)	0.028 (2)	0.039 (3)	0.0062 (18)	0.0128 (19)	−0.0004 (18)
O12	0.0134 (17)	0.0238 (17)	0.0219 (19)	−0.0028 (16)	0.0084 (15)	−0.0012 (15)
C11	0.029 (3)	0.027 (3)	0.015 (3)	−0.003 (3)	0.001 (2)	0.004 (2)
C12	0.028 (3)	0.026 (3)	0.018 (3)	−0.003 (3)	0.000 (2)	0.001 (2)
C13	0.030 (3)	0.033 (3)	0.026 (3)	−0.007 (3)	0.003 (3)	−0.007 (2)
C14	0.037 (4)	0.031 (3)	0.044 (4)	−0.011 (3)	0.012 (3)	−0.007 (3)
C15	0.050 (4)	0.028 (3)	0.056 (4)	0.002 (3)	0.008 (4)	−0.004 (3)
C16	0.035 (4)	0.027 (3)	0.041 (4)	0.006 (3)	0.009 (3)	−0.001 (3)
C17	0.019 (3)	0.031 (3)	0.025 (3)	0.001 (3)	0.004 (2)	−0.001 (2)
C18	0.018 (3)	0.027 (3)	0.021 (3)	0.002 (2)	0.001 (2)	0.005 (2)
C19	0.023 (3)	0.027 (3)	0.020 (3)	−0.003 (2)	−0.002 (2)	−0.001 (2)
C21	0.025 (3)	0.028 (3)	0.035 (3)	−0.004 (3)	0.004 (3)	−0.011 (3)
C22	0.022 (3)	0.022 (3)	0.040 (4)	0.001 (2)	−0.003 (3)	−0.010 (2)
C23	0.024 (3)	0.035 (3)	0.052 (4)	0.001 (3)	−0.008 (3)	−0.011 (3)
C24	0.032 (4)	0.039 (3)	0.058 (5)	−0.004 (3)	−0.018 (3)	−0.013 (3)
C25	0.045 (4)	0.036 (3)	0.039 (4)	−0.001 (3)	−0.011 (3)	−0.007 (3)
C26	0.037 (4)	0.029 (3)	0.044 (4)	−0.002 (3)	0.003 (3)	−0.006 (3)
C27	0.018 (3)	0.024 (3)	0.048 (4)	−0.004 (2)	0.007 (3)	−0.011 (3)
C28	0.020 (3)	0.020 (3)	0.032 (3)	−0.001 (2)	0.007 (3)	−0.004 (2)
C29	0.020 (3)	0.020 (3)	0.031 (3)	−0.003 (2)	0.010 (3)	−0.001 (2)
C31	0.023 (3)	0.032 (3)	0.019 (3)	0.003 (3)	−0.001 (2)	0.002 (2)
C32	0.023 (3)	0.029 (3)	0.020 (3)	0.002 (3)	0.003 (2)	0.005 (2)
C33	0.023 (3)	0.041 (3)	0.028 (3)	0.002 (3)	0.002 (3)	−0.003 (3)
C34	0.025 (3)	0.043 (3)	0.032 (3)	0.010 (3)	0.004 (3)	−0.008 (3)
C35	0.033 (4)	0.032 (3)	0.036 (4)	0.006 (3)	0.005 (3)	−0.003 (3)
C36	0.029 (3)	0.023 (3)	0.039 (4)	0.000 (3)	0.010 (3)	0.003 (2)
C37	0.012 (3)	0.035 (3)	0.028 (3)	−0.005 (3)	0.005 (2)	0.007 (2)
C38	0.020 (3)	0.026 (3)	0.016 (3)	0.000 (2)	0.007 (2)	0.000 (2)
C39	0.019 (3)	0.022 (3)	0.011 (2)	−0.004 (2)	0.003 (2)	0.003 (2)
C41	0.025 (3)	0.023 (3)	0.032 (3)	−0.001 (2)	0.003 (3)	−0.005 (2)
C42	0.020 (3)	0.027 (3)	0.030 (3)	−0.002 (2)	0.008 (3)	−0.004 (2)
C43	0.019 (3)	0.029 (3)	0.040 (4)	−0.002 (2)	0.006 (3)	−0.003 (3)
C44	0.031 (4)	0.040 (3)	0.033 (4)	−0.003 (3)	0.018 (3)	0.003 (3)
C45	0.031 (3)	0.043 (3)	0.030 (3)	0.002 (3)	0.011 (3)	0.000 (3)
C46	0.026 (3)	0.032 (3)	0.030 (3)	−0.008 (3)	0.001 (3)	−0.003 (2)
C47	0.016 (3)	0.025 (3)	0.034 (3)	−0.004 (2)	0.001 (3)	−0.004 (2)
C48	0.022 (3)	0.023 (3)	0.028 (3)	0.000 (2)	−0.001 (2)	−0.007 (2)
C49	0.020 (3)	0.016 (2)	0.027 (3)	−0.002 (2)	−0.003 (2)	−0.001 (2)
C110	0.023 (3)	0.023 (3)	0.025 (3)	0.002 (2)	0.006 (2)	0.001 (2)
C111	0.028 (3)	0.035 (3)	0.027 (3)	0.004 (3)	0.005 (3)	0.002 (2)
C112	0.019 (3)	0.040 (3)	0.033 (3)	0.011 (3)	0.010 (3)	0.008 (3)
C113	0.023 (3)	0.032 (3)	0.030 (3)	0.001 (3)	0.009 (3)	0.006 (2)
C114	0.042 (4)	0.032 (3)	0.060 (4)	0.008 (3)	0.017 (4)	−0.008 (3)
C115	0.032 (3)	0.052 (4)	0.028 (3)	0.005 (3)	0.015 (3)	0.007 (3)
C210	0.020 (3)	0.024 (3)	0.038 (3)	0.003 (2)	0.010 (3)	0.001 (2)
C211	0.035 (4)	0.027 (3)	0.042 (4)	0.006 (3)	0.018 (3)	0.005 (3)
C212	0.028 (3)	0.025 (3)	0.053 (4)	−0.003 (3)	0.020 (3)	0.007 (3)
C213	0.023 (3)	0.026 (3)	0.050 (4)	−0.005 (3)	0.014 (3)	−0.001 (3)
C214	0.051 (4)	0.034 (3)	0.048 (4)	−0.003 (3)	0.019 (3)	0.011 (3)
C215	0.056 (5)	0.062 (5)	0.112 (7)	0.004 (4)	0.006 (5)	−0.026 (5)
C310	0.021 (3)	0.021 (3)	0.021 (3)	−0.001 (2)	0.003 (2)	0.002 (2)
C311	0.032 (3)	0.026 (3)	0.018 (3)	−0.012 (3)	0.005 (3)	0.002 (2)
C312	0.036 (4)	0.033 (3)	0.029 (3)	−0.016 (3)	−0.003 (3)	0.003 (3)
C313	0.023 (3)	0.039 (3)	0.022 (3)	−0.007 (3)	−0.001 (3)	0.008 (2)
C314	0.041 (4)	0.028 (3)	0.048 (4)	−0.010 (3)	0.001 (3)	−0.010 (3)
C315	0.025 (3)	0.075 (4)	0.032 (4)	−0.002 (3)	−0.003 (3)	0.001 (3)
C410	0.022 (3)	0.018 (3)	0.027 (3)	0.002 (2)	0.003 (2)	−0.006 (2)
C411	0.023 (3)	0.015 (2)	0.038 (3)	0.000 (2)	−0.004 (3)	−0.003 (2)
C412	0.017 (3)	0.033 (3)	0.044 (4)	−0.002 (3)	−0.006 (3)	−0.007 (3)
C413	0.016 (3)	0.034 (3)	0.041 (4)	−0.003 (3)	0.005 (3)	−0.003 (3)
C414	0.032 (3)	0.030 (3)	0.038 (3)	0.004 (3)	−0.011 (3)	−0.006 (3)
C415	0.059 (5)	0.047 (4)	0.073 (5)	0.023 (4)	0.033 (4)	0.010 (4)
O1	0.112 (7)	0.108 (5)	0.122 (7)	0.010 (5)	0.007 (6)	0.021 (5)
C1	0.057 (7)	0.043 (5)	0.138 (10)	0.012 (5)	0.023 (7)	0.001 (6)

### Geometric parameters (Å, º)

**Table:**

Ni1—O11	1.975 (3)	C33—H33	0.9300
Ni1—N11	1.976 (4)	C34—C35	1.386 (7)
Ni1—O42	2.072 (3)	C34—H34	0.9300
Ni1—O12	2.083 (3)	C35—C36	1.361 (7)
Ni1—O13	2.090 (4)	C35—H35	0.9300
Ni1—O32	2.211 (3)	C36—H36	0.9300
Ni2—O21	1.965 (4)	C37—H37	0.9300
Ni2—N21	1.969 (4)	C38—C313	1.390 (7)
Ni2—O12	2.034 (3)	C38—C39	1.394 (6)
Ni2—O22	2.043 (3)	C39—C310	1.396 (6)
Ni2—O23	2.137 (4)	C41—C46	1.415 (7)
Ni2—O42	2.247 (3)	C41—C42	1.446 (7)
Ni3—N31	1.967 (4)	C42—C43	1.404 (7)
Ni3—O31	1.969 (3)	C42—C47	1.431 (7)
Ni3—O22	2.049 (3)	C43—C44	1.360 (7)
Ni3—O32	2.053 (3)	C43—H43	0.9300
Ni3—O33	2.071 (4)	C44—C45	1.401 (8)
Ni3—O12	2.194 (3)	C44—H44	0.9300
Ni4—O41	1.957 (3)	C45—C46	1.360 (7)
Ni4—N41	1.971 (4)	C45—H45	0.9300
Ni4—O42	2.049 (3)	C46—H46	0.9300
Ni4—O32	2.067 (3)	C47—H47	0.9300
Ni4—O43	2.103 (3)	C48—C413	1.384 (7)
Ni4—O22	2.219 (3)	C48—C49	1.427 (7)
N11—C17	1.285 (6)	C49—C410	1.382 (7)
N11—C18	1.427 (6)	C110—C111	1.400 (7)
N21—C27	1.285 (6)	C110—H110	0.9300
N21—C28	1.413 (6)	C111—C112	1.393 (7)
N31—C37	1.284 (6)	C111—C114	1.517 (7)
N31—C38	1.425 (6)	C112—C113	1.377 (6)
N41—C47	1.285 (6)	C112—H112	0.9300
N41—C48	1.422 (6)	C113—H113	0.9300
O11—C11	1.328 (6)	C114—H11A	0.9600
O13—C115	1.427 (6)	C114—H11B	0.9600
O13—H13O	0.836 (19)	C114—H11C	0.9600
O21—C21	1.318 (6)	C115—H11D	0.9600
O22—C29	1.371 (5)	C115—H11E	0.9600
O23—C215	1.446 (7)	C115—H11F	0.9600
O23—H23O	0.84 (2)	C210—C211	1.386 (7)
O31—C31	1.310 (6)	C210—H210	0.9300
O32—C39	1.365 (5)	C211—C212	1.390 (8)
O33—C315	1.414 (6)	C211—C214	1.505 (7)
O33—H33O	0.82 (2)	C212—C213	1.367 (7)
O41—C41	1.310 (6)	C212—H212	0.9300
O42—C49	1.359 (6)	C213—H213	0.9300
O43—C415	1.391 (6)	C214—H21A	0.9600
O43—H43O	0.81 (2)	C214—H21B	0.9600
O12—C19	1.364 (5)	C214—H21C	0.9600
C11—C16	1.391 (6)	C215—H21D	0.9600
C11—C12	1.424 (7)	C215—H21E	0.9600
C12—C13	1.413 (7)	C215—H21F	0.9600
C12—C17	1.438 (6)	C310—C311	1.381 (7)
C13—C14	1.345 (7)	C310—H310	0.9300
C13—H13	0.9300	C311—C312	1.379 (7)
C14—C15	1.392 (8)	C311—C314	1.505 (6)
C14—H14	0.9300	C312—C313	1.379 (7)
C15—C16	1.377 (7)	C312—H312	0.9300
C15—H15	0.9300	C313—H313	0.9300
C16—H16	0.9300	C314—H31A	0.9600
C17—H17	0.9300	C314—H31B	0.9600
C18—C113	1.377 (6)	C314—H31C	0.9600
C18—C19	1.408 (6)	C315—H31D	0.9600
C19—C110	1.374 (6)	C315—H31E	0.9600
C21—C26	1.407 (7)	C315—H31F	0.9600
C21—C22	1.412 (7)	C410—C411	1.399 (7)
C22—C23	1.410 (7)	C410—H410	0.9300
C22—C27	1.458 (7)	C411—C412	1.393 (7)
C23—C24	1.364 (8)	C411—C414	1.496 (7)
C23—H23	0.9300	C412—C413	1.369 (7)
C24—C25	1.376 (8)	C412—H412	0.9300
C24—H24	0.9300	C413—H413	0.9300
C25—C26	1.378 (8)	C414—H41A	0.9600
C25—H25	0.9300	C414—H41B	0.9600
C26—H26	0.9300	C414—H41C	0.9600
C27—H27	0.9300	C415—H41D	0.9600
C28—C213	1.388 (7)	C415—H41E	0.9600
C28—C29	1.410 (7)	C415—H41F	0.9600
C29—C210	1.393 (7)	O1—C1	1.383 (9)
C31—C36	1.413 (6)	O1—H11O	0.827 (8)
C31—C32	1.427 (7)	C1—H1A	0.9600
C32—C33	1.413 (7)	C1—H1B	0.9600
C32—C37	1.444 (6)	C1—H1C	0.9600
C33—C34	1.382 (7)		
			
O11—Ni1—N11	93.97 (15)	C29—C28—N21	115.7 (4)
O11—Ni1—O42	98.72 (13)	O22—C29—C210	122.8 (5)
N11—Ni1—O42	167.26 (14)	O22—C29—C28	117.6 (5)
O11—Ni1—O12	168.32 (14)	C210—C29—C28	119.6 (5)
N11—Ni1—O12	81.52 (14)	O31—C31—C36	119.0 (5)
O42—Ni1—O12	85.81 (12)	O31—C31—C32	124.7 (4)
O11—Ni1—O13	101.58 (13)	C36—C31—C32	116.3 (5)
N11—Ni1—O13	92.06 (15)	C33—C32—C31	119.1 (5)
O42—Ni1—O13	86.55 (13)	C33—C32—C37	115.7 (5)
O12—Ni1—O13	89.38 (13)	C31—C32—C37	125.1 (5)
O11—Ni1—O32	91.02 (12)	C34—C33—C32	122.3 (5)
N11—Ni1—O32	96.28 (14)	C34—C33—H33	118.8
O42—Ni1—O32	82.40 (12)	C32—C33—H33	118.8
O12—Ni1—O32	78.86 (12)	C33—C34—C35	118.1 (5)
O13—Ni1—O32	164.38 (12)	C33—C34—H34	120.9
O21—Ni2—N21	94.24 (16)	C35—C34—H34	120.9
O21—Ni2—O12	97.17 (13)	C36—C35—C34	121.1 (5)
N21—Ni2—O12	168.59 (16)	C36—C35—H35	119.4
O21—Ni2—O22	170.86 (14)	C34—C35—H35	119.4
N21—Ni2—O22	83.23 (15)	C35—C36—C31	123.0 (5)
O12—Ni2—O22	85.45 (13)	C35—C36—H36	118.5
O21—Ni2—O23	100.18 (14)	C31—C36—H36	118.5
N21—Ni2—O23	90.40 (15)	N31—C37—C32	124.8 (5)
O12—Ni2—O23	87.82 (13)	N31—C37—H37	117.6
O22—Ni2—O23	88.64 (14)	C32—C37—H37	117.6
O21—Ni2—O42	93.72 (13)	C313—C38—C39	119.3 (4)
N21—Ni2—O42	96.52 (14)	C313—C38—N31	125.7 (5)
O12—Ni2—O42	82.54 (12)	C39—C38—N31	114.9 (4)
O22—Ni2—O42	77.90 (12)	O32—C39—C38	118.0 (4)
O23—Ni2—O42	163.98 (13)	O32—C39—C310	122.9 (4)
N31—Ni3—O31	94.13 (15)	C38—C39—C310	119.1 (4)
N31—Ni3—O22	168.96 (14)	O41—C41—C46	119.1 (5)
O31—Ni3—O22	96.86 (13)	O41—C41—C42	124.7 (5)
N31—Ni3—O32	82.19 (14)	C46—C41—C42	116.2 (5)
O31—Ni3—O32	172.48 (13)	C43—C42—C47	117.3 (5)
O22—Ni3—O32	86.78 (12)	C43—C42—C41	118.6 (5)
N31—Ni3—O33	92.10 (16)	C47—C42—C41	124.1 (5)
O31—Ni3—O33	98.53 (14)	C44—C43—C42	123.4 (5)
O22—Ni3—O33	87.24 (14)	C44—C43—H43	118.3
O32—Ni3—O33	88.19 (13)	C42—C43—H43	118.3
N31—Ni3—O12	96.99 (14)	C43—C44—C45	117.8 (5)
O31—Ni3—O12	94.12 (13)	C43—C44—H44	121.1
O22—Ni3—O12	81.29 (12)	C45—C44—H44	121.1
O32—Ni3—O12	79.88 (12)	C46—C45—C44	121.5 (6)
O33—Ni3—O12	163.85 (13)	C46—C45—H45	119.3
O41—Ni4—N41	93.86 (16)	C44—C45—H45	119.3
O41—Ni4—O42	171.08 (13)	C45—C46—C41	122.4 (5)
N41—Ni4—O42	82.65 (15)	C45—C46—H46	118.8
O41—Ni4—O32	96.76 (14)	C41—C46—H46	118.8
N41—Ni4—O32	169.24 (15)	N41—C47—C42	125.6 (5)
O42—Ni4—O32	86.60 (12)	N41—C47—H47	117.2
O41—Ni4—O43	99.72 (14)	C42—C47—H47	117.2
N41—Ni4—O43	92.60 (15)	C413—C48—N41	125.8 (5)
O42—Ni4—O43	88.67 (13)	C413—C48—C49	119.4 (5)
O32—Ni4—O43	87.31 (13)	N41—C48—C49	114.7 (4)
O41—Ni4—O22	93.77 (13)	O42—C49—C410	123.5 (5)
N41—Ni4—O22	95.49 (14)	O42—C49—C48	117.5 (4)
O42—Ni4—O22	78.46 (12)	C410—C49—C48	118.9 (5)
O32—Ni4—O22	82.13 (12)	C19—C110—C111	122.4 (5)
O43—Ni4—O22	163.76 (13)	C19—C110—H110	118.8
C17—N11—C18	123.6 (4)	C111—C110—H110	118.8
C17—N11—Ni1	125.1 (3)	C112—C111—C110	117.8 (5)
C18—N11—Ni1	111.3 (3)	C112—C111—C114	120.7 (5)
C27—N21—C28	124.4 (5)	C110—C111—C114	121.4 (5)
C27—N21—Ni2	125.5 (4)	C113—C112—C111	120.6 (5)
C28—N21—Ni2	110.0 (3)	C113—C112—H112	119.7
C37—N31—C38	123.4 (4)	C111—C112—H112	119.7
C37—N31—Ni3	125.5 (3)	C112—C113—C18	120.8 (5)
C38—N31—Ni3	111.1 (3)	C112—C113—H113	119.6
C47—N41—C48	123.9 (4)	C18—C113—H113	119.6
C47—N41—Ni4	124.9 (4)	C111—C114—H11A	109.5
C48—N41—Ni4	111.0 (3)	C111—C114—H11B	109.5
C11—O11—Ni1	124.8 (3)	H11A—C114—H11B	109.5
C115—O13—Ni1	128.6 (3)	C111—C114—H11C	109.5
C115—O13—H13O	108 (4)	H11A—C114—H11C	109.5
Ni1—O13—H13O	111 (5)	H11B—C114—H11C	109.5
C21—O21—Ni2	124.5 (3)	O13—C115—H11D	109.5
C29—O22—Ni2	107.1 (3)	O13—C115—H11E	109.5
C29—O22—Ni3	136.7 (3)	H11D—C115—H11E	109.5
Ni2—O22—Ni3	97.63 (13)	O13—C115—H11F	109.5
C29—O22—Ni4	115.7 (3)	H11D—C115—H11F	109.5
Ni2—O22—Ni4	102.38 (13)	H11E—C115—H11F	109.5
Ni3—O22—Ni4	92.11 (12)	C211—C210—C29	121.0 (5)
C215—O23—Ni2	128.3 (4)	C211—C210—H210	119.5
C215—O23—H23O	120 (5)	C29—C210—H210	119.5
Ni2—O23—H23O	97 (4)	C210—C211—C212	118.4 (5)
C31—O31—Ni3	124.4 (3)	C210—C211—C214	120.1 (6)
C39—O32—Ni3	108.0 (3)	C212—C211—C214	121.6 (5)
C39—O32—Ni4	138.3 (3)	C213—C212—C211	121.7 (5)
Ni3—O32—Ni4	96.56 (12)	C213—C212—H212	119.2
C39—O32—Ni1	114.7 (3)	C211—C212—H212	119.2
Ni3—O32—Ni1	100.82 (12)	C212—C213—C28	120.6 (6)
Ni4—O32—Ni1	92.08 (13)	C212—C213—H213	119.7
C315—O33—Ni3	128.3 (3)	C28—C213—H213	119.7
C315—O33—H33O	110 (5)	C211—C214—H21A	109.5
Ni3—O33—H33O	110 (5)	C211—C214—H21B	109.5
C41—O41—Ni4	124.6 (3)	H21A—C214—H21B	109.5
C49—O42—Ni4	108.8 (3)	C211—C214—H21C	109.5
C49—O42—Ni1	139.4 (3)	H21A—C214—H21C	109.5
Ni4—O42—Ni1	96.81 (14)	H21B—C214—H21C	109.5
C49—O42—Ni2	112.7 (3)	O23—C215—H21D	109.5
Ni4—O42—Ni2	101.23 (13)	O23—C215—H21E	109.5
Ni1—O42—Ni2	91.58 (12)	H21D—C215—H21E	109.5
C415—O43—Ni4	128.5 (3)	O23—C215—H21F	109.5
C415—O43—H43O	110 (5)	H21D—C215—H21F	109.5
Ni4—O43—H43O	113 (4)	H21E—C215—H21F	109.5
C19—O12—Ni2	135.6 (3)	C311—C310—C39	121.4 (5)
C19—O12—Ni1	107.6 (3)	C311—C310—H310	119.3
Ni2—O12—Ni1	97.62 (13)	C39—C310—H310	119.3
C19—O12—Ni3	116.3 (3)	C312—C311—C310	118.7 (5)
Ni2—O12—Ni3	93.45 (12)	C312—C311—C314	121.1 (5)
Ni1—O12—Ni3	100.42 (12)	C310—C311—C314	120.2 (5)
O11—C11—C16	118.7 (5)	C311—C312—C313	121.1 (5)
O11—C11—C12	124.5 (4)	C311—C312—H312	119.5
C16—C11—C12	116.8 (5)	C313—C312—H312	119.5
C13—C12—C11	118.8 (5)	C312—C313—C38	120.4 (5)
C13—C12—C17	116.1 (5)	C312—C313—H313	119.8
C11—C12—C17	125.1 (5)	C38—C313—H313	119.8
C14—C13—C12	122.8 (5)	C311—C314—H31A	109.5
C14—C13—H13	118.6	C311—C314—H31B	109.5
C12—C13—H13	118.6	H31A—C314—H31B	109.5
C13—C14—C15	118.7 (5)	C311—C314—H31C	109.5
C13—C14—H14	120.6	H31A—C314—H31C	109.5
C15—C14—H14	120.6	H31B—C314—H31C	109.5
C16—C15—C14	120.2 (5)	O33—C315—H31D	109.5
C16—C15—H15	119.9	O33—C315—H31E	109.5
C14—C15—H15	119.9	H31D—C315—H31E	109.5
C15—C16—C11	122.7 (5)	O33—C315—H31F	109.5
C15—C16—H16	118.6	H31D—C315—H31F	109.5
C11—C16—H16	118.6	H31E—C315—H31F	109.5
N11—C17—C12	125.6 (5)	C49—C410—C411	121.4 (5)
N11—C17—H17	117.2	C49—C410—H410	119.3
C12—C17—H17	117.2	C411—C410—H410	119.3
C113—C18—C19	120.1 (5)	C412—C411—C410	118.2 (5)
C113—C18—N11	125.1 (4)	C412—C411—C414	122.1 (5)
C19—C18—N11	114.7 (4)	C410—C411—C414	119.7 (5)
O12—C19—C110	123.6 (4)	C413—C412—C411	121.7 (5)
O12—C19—C18	118.1 (4)	C413—C412—H412	119.1
C110—C19—C18	118.3 (5)	C411—C412—H412	119.1
O21—C21—C26	117.8 (5)	C412—C413—C48	120.3 (5)
O21—C21—C22	125.3 (5)	C412—C413—H413	119.8
C26—C21—C22	116.9 (5)	C48—C413—H413	119.8
C23—C22—C21	119.3 (6)	C411—C414—H41A	109.5
C23—C22—C27	116.0 (5)	C411—C414—H41B	109.5
C21—C22—C27	124.6 (5)	H41A—C414—H41B	109.5
C24—C23—C22	122.0 (6)	C411—C414—H41C	109.5
C24—C23—H23	119.0	H41A—C414—H41C	109.5
C22—C23—H23	119.0	H41B—C414—H41C	109.5
C23—C24—C25	119.1 (6)	O43—C415—H41D	109.5
C23—C24—H24	120.4	O43—C415—H41E	109.5
C25—C24—H24	120.4	H41D—C415—H41E	109.5
C24—C25—C26	120.5 (6)	O43—C415—H41F	109.5
C24—C25—H25	119.8	H41D—C415—H41F	109.5
C26—C25—H25	119.8	H41E—C415—H41F	109.5
C25—C26—C21	122.2 (6)	C1—O1—H11O	109.0 (9)
C25—C26—H26	118.9	O1—C1—H1A	109.5
C21—C26—H26	118.9	O1—C1—H1B	109.5
N21—C27—C22	125.0 (5)	H1A—C1—H1B	109.5
N21—C27—H27	117.5	O1—C1—H1C	109.5
C22—C27—H27	117.5	H1A—C1—H1C	109.5
C213—C28—C29	118.8 (5)	H1B—C1—H1C	109.5
C213—C28—N21	125.3 (5)		
			
Ni1—O11—C11—C16	177.5 (3)	Ni3—N31—C38—C313	−165.5 (4)
Ni1—O11—C11—C12	−2.8 (7)	C37—N31—C38—C39	−171.0 (5)
O11—C11—C12—C13	179.1 (4)	Ni3—N31—C38—C39	10.9 (5)
C16—C11—C12—C13	−1.2 (7)	Ni3—O32—C39—C38	−21.8 (5)
O11—C11—C12—C17	−3.9 (8)	Ni4—O32—C39—C38	−145.0 (4)
C16—C11—C12—C17	175.8 (5)	Ni1—O32—C39—C38	89.7 (4)
C11—C12—C13—C14	0.6 (8)	Ni3—O32—C39—C310	160.1 (4)
C17—C12—C13—C14	−176.7 (5)	Ni4—O32—C39—C310	37.0 (7)
C12—C13—C14—C15	0.5 (9)	Ni1—O32—C39—C310	−88.3 (4)
C13—C14—C15—C16	−1.1 (9)	C313—C38—C39—O32	−175.2 (4)
C14—C15—C16—C11	0.5 (9)	N31—C38—C39—O32	8.1 (6)
O11—C11—C16—C15	−179.6 (5)	C313—C38—C39—C310	2.9 (7)
C12—C11—C16—C15	0.7 (8)	N31—C38—C39—C310	−173.7 (4)
C18—N11—C17—C12	−173.6 (5)	Ni4—O41—C41—C46	−169.9 (3)
Ni1—N11—C17—C12	7.3 (7)	Ni4—O41—C41—C42	9.6 (7)
C13—C12—C17—N11	178.5 (5)	O41—C41—C42—C43	−175.7 (4)
C11—C12—C17—N11	1.4 (8)	C46—C41—C42—C43	3.8 (7)
C17—N11—C18—C113	20.7 (8)	O41—C41—C42—C47	2.8 (8)
Ni1—N11—C18—C113	−160.2 (4)	C46—C41—C42—C47	−177.6 (4)
C17—N11—C18—C19	−165.0 (5)	C47—C42—C43—C44	178.8 (5)
Ni1—N11—C18—C19	14.1 (5)	C41—C42—C43—C44	−2.5 (8)
Ni2—O12—C19—C110	36.8 (7)	C42—C43—C44—C45	0.1 (8)
Ni1—O12—C19—C110	158.3 (4)	C43—C44—C45—C46	0.9 (8)
Ni3—O12—C19—C110	−90.1 (5)	C44—C45—C46—C41	0.6 (8)
Ni2—O12—C19—C18	−144.0 (4)	O41—C41—C46—C45	176.6 (4)
Ni1—O12—C19—C18	−22.5 (5)	C42—C41—C46—C45	−3.0 (7)
Ni3—O12—C19—C18	89.1 (5)	C48—N41—C47—C42	178.2 (4)
C113—C18—C19—O12	−178.7 (5)	Ni4—N41—C47—C42	−7.0 (7)
N11—C18—C19—O12	6.7 (7)	C43—C42—C47—N41	174.3 (4)
C113—C18—C19—C110	0.5 (7)	C41—C42—C47—N41	−4.3 (8)
N11—C18—C19—C110	−174.1 (4)	C47—N41—C48—C413	−17.4 (7)
Ni2—O21—C21—C26	−172.2 (3)	Ni4—N41—C48—C413	167.2 (4)
Ni2—O21—C21—C22	9.3 (7)	C47—N41—C48—C49	163.4 (4)
O21—C21—C22—C23	179.6 (5)	Ni4—N41—C48—C49	−12.0 (5)
C26—C21—C22—C23	1.2 (7)	Ni4—O42—C49—C410	−163.9 (4)
O21—C21—C22—C27	−0.9 (8)	Ni1—O42—C49—C410	−37.8 (7)
C26—C21—C22—C27	−179.4 (5)	Ni2—O42—C49—C410	84.6 (5)
C21—C22—C23—C24	−0.2 (8)	Ni4—O42—C49—C48	19.9 (5)
C27—C22—C23—C24	−179.6 (5)	Ni1—O42—C49—C48	146.0 (4)
C22—C23—C24—C25	−0.7 (8)	Ni2—O42—C49—C48	−91.6 (4)
C23—C24—C25—C26	0.6 (8)	C413—C48—C49—O42	174.8 (4)
C24—C25—C26—C21	0.5 (8)	N41—C48—C49—O42	−6.0 (6)
O21—C21—C26—C25	−179.9 (5)	C413—C48—C49—C410	−1.5 (7)
C22—C21—C26—C25	−1.3 (8)	N41—C48—C49—C410	177.7 (4)
C28—N21—C27—C22	175.1 (4)	O12—C19—C110—C111	177.8 (5)
Ni2—N21—C27—C22	−0.1 (7)	C18—C19—C110—C111	−1.4 (8)
C23—C22—C27—N21	175.2 (5)	C19—C110—C111—C112	0.7 (8)
C21—C22—C27—N21	−4.2 (8)	C19—C110—C111—C114	−178.0 (5)
C27—N21—C28—C213	−12.7 (8)	C110—C111—C112—C113	1.0 (8)
Ni2—N21—C28—C213	163.1 (4)	C114—C111—C112—C113	179.7 (5)
C27—N21—C28—C29	172.4 (4)	C111—C112—C113—C18	−1.8 (8)
Ni2—N21—C28—C29	−11.8 (5)	C19—C18—C113—C112	1.1 (8)
Ni2—O22—C29—C210	−158.7 (4)	N11—C18—C113—C112	175.1 (5)
Ni3—O22—C29—C210	−36.8 (7)	O22—C29—C210—C211	−176.8 (4)
Ni4—O22—C29—C210	88.0 (5)	C28—C29—C210—C211	1.6 (7)
Ni2—O22—C29—C28	22.9 (5)	C29—C210—C211—C212	0.3 (7)
Ni3—O22—C29—C28	144.8 (4)	C29—C210—C211—C214	−179.6 (5)
Ni4—O22—C29—C28	−90.5 (4)	C210—C211—C212—C213	−1.8 (8)
C213—C28—C29—O22	176.5 (4)	C214—C211—C212—C213	178.1 (5)
N21—C28—C29—O22	−8.3 (6)	C211—C212—C213—C28	1.4 (8)
C213—C28—C29—C210	−2.1 (7)	C29—C28—C213—C212	0.6 (7)
N21—C28—C29—C210	173.2 (4)	N21—C28—C213—C212	−174.1 (5)
Ni3—O31—C31—C36	169.8 (4)	O32—C39—C310—C311	175.4 (4)
Ni3—O31—C31—C32	−10.2 (7)	C38—C39—C310—C311	−2.7 (7)
O31—C31—C32—C33	180.0 (5)	C39—C310—C311—C312	0.6 (7)
C36—C31—C32—C33	0.0 (7)	C39—C310—C311—C314	−177.8 (4)
O31—C31—C32—C37	−0.1 (8)	C310—C311—C312—C313	1.2 (8)
C36—C31—C32—C37	179.8 (5)	C314—C311—C312—C313	179.6 (5)
C31—C32—C33—C34	0.3 (8)	C311—C312—C313—C38	−0.9 (8)
C37—C32—C33—C34	−179.6 (5)	C39—C38—C313—C312	−1.2 (7)
C32—C33—C34—C35	−0.1 (8)	N31—C38—C313—C312	175.1 (4)
C33—C34—C35—C36	−0.2 (8)	O42—C49—C410—C411	−173.0 (4)
C34—C35—C36—C31	0.4 (9)	C48—C49—C410—C411	3.1 (7)
O31—C31—C36—C35	179.7 (5)	C49—C410—C411—C412	−2.1 (7)
C32—C31—C36—C35	−0.3 (8)	C49—C410—C411—C414	176.6 (4)
C38—N31—C37—C32	−175.3 (4)	C410—C411—C412—C413	−0.6 (7)
Ni3—N31—C37—C32	2.4 (7)	C414—C411—C412—C413	−179.2 (5)
C33—C32—C37—N31	−175.6 (5)	C411—C412—C413—C48	2.1 (8)
C31—C32—C37—N31	4.5 (8)	N41—C48—C413—C412	179.8 (4)
C37—N31—C38—C313	12.5 (8)	C49—C48—C413—C412	−1.0 (7)

### Hydrogen-bond geometry (Å, º)

**Table:**

*D*—H···*A*	*D*—H	H···*A*	*D*···*A*	*D*—H···*A*
O13—H13*O*···O21	0.84 (2)	1.89 (3)	2.688 (5)	161 (6)
O23—H23*O*···O31	0.84 (2)	1.87 (2)	2.709 (5)	177 (7)
O33—H33*O*···O41	0.82 (2)	1.88 (3)	2.645 (5)	155 (7)
O43—H43*O*···O11	0.81 (2)	1.93 (3)	2.686 (5)	155 (7)
C46—H46···O11^i^	0.93	2.55	3.307 (6)	139
C110—H110···O23	0.93	2.42	3.177 (6)	138
C210—H210···O33	0.93	2.44	3.175 (6)	136
C310—H310···O43	0.93	2.48	3.218 (6)	137
C410—H410···O13	0.93	2.45	3.189 (6)	136
O1—H11*O*···O31	0.83	2.2100	3.034 (8)	177

Symmetry code: (i) *x*, −*y*+1/2, *z*−1/2.

## References

[bb1] Ballester, L., Coronado, E., Gutierrez, A., Monge, A., Perpinan, M. F., Pinilla, E. & Rico, T. (1992). *Inorg. Chem.* **31**, 2053–2056.

[bb2] Bertrand, J. A., Marbella, C. & Vanderveer, D. G. (1978). *Inorg. Chim. Acta*, **26**, 113–118.

[bb3] Cindrić, M., Pavlović, G., Pajić, D., Zadro, K., Cinčić, D., Hrenar, T., Lekšić, E., Pinar Prieto, A. B., Lazić, P. & Šišak Jung, D. (2016). *New J. Chem.* **40**, 6604–6614.

[bb4] Cotton, F. A., Herrero, S., Jiménez-Aparicio, R., Murillo, C. A., Urbanos, F. A., Villagrán, D. & Wang, X. (2007). *J. Am. Chem. Soc.* **129**, 12666–12667. 10.1021/ja075808z 17910458

[bb5] Gladfelter, W. L., Lynch, M. W., Schaefer, W. P., Hendrickson, D. N. & Gray, H. B. (1981). *Inorg. Chem.* **20**, 2390–2397.

[bb6] Halcrow, M. A., Sun, J. S., Huffman, J. C. & Christou, G. (1995). *Inorg. Chem.* **34**, 4167–4177.

[bb7] Ji, C. M., Yang, H. J., Zhao, C. C., Tangoulis, V., Cui, A. L. & Kou, H. Z. (2009). *Cryst. Growth Des.* **9**, 4607–4609.

[bb8] Karmakar, S. & Khanra, S. (2014). *CrystEngComm*, **16**, 2371–2383.

[bb9] Kou, H. Z., An, G. Y., Ji, C. M., Wang, B. W. & Cui, A. L. (2010). *Dalton Trans.* **39**, 9604–9610. 10.1039/c0dt00528b 20820613

[bb10] Lawrence, J., Yang, E.-C., Edwards, R., Olmstead, M. M., Ramsey, C., Dalal, N. S., Gantzel, P. K., Hill, S. & Hendrickson, D. N. (2008). *Inorg. Chem.* **47**, 1965–1974. 10.1021/ic701416w 18284196

[bb11] Macrae, C. F., Edgington, P. R., McCabe, P., Pidcock, E., Shields, G. P., Taylor, R., Towler, M. & van de Streek, J. (2006). *J. Appl. Cryst.* **39**, 453–457.

[bb12] Osa, S., Kido, T., Matsumoto, N., Re, N., Pochaba, A. & Mrozinski, J. (2004). *J. Am. Chem. Soc.* **126**, 420–421. 10.1021/ja037365e 14719911

[bb13] Oxford Diffraction (2010). *CrysAlis PRO*. Oxford Diffraction Ltd, Yarnton, England.

[bb14] Papatriantafyllopoulou, C., Jones, L. F., Nguyen, T. D., Matamoros-Salvador, N., Cunha-Silva, L., Almeida Paz, F. A., Rocha, J., Evangelisti, M., Brechin, E. K. & Perlepes, S. P. (2008). *Dalton Trans.* pp. 3153. 10.1039/b802927j 18688412

[bb15] Pardo, E., Ruiz-Garcia, R., Cano, J., Ottenwaelder, X., Lescouëzec, R., Journaux, Y., Lloret, F. & Julve, M. (2008). *Dalton Trans.* pp. 2780–2805. 10.1039/b801222a 18478138

[bb16] Perlepe, P. S., Athanasopoulou, A. A., Alexopoulou, K. I., Raptopoulou, C. P., Psycharis, V., Escuer, A., Perlepes, S. P. & Stamatatos, T. C. (2014). *Dalton Trans.* **43**, 16605–16609. 10.1039/c4dt02434f 25307956

[bb17] Petit, S., Neugebauer, P., Pilet, G. M., Chastanet, G., Barra, A. L., Antunes, A. B., Wernsdorfer, W. & Luneau, D. (2012). *Inorg. Chem.* **51**, 6645–6654. 10.1021/ic3001637 22662964

[bb18] Polyakov, A. O., Arkenbout, A. H., Baas, J., Blake, R. G., Meetsma, A., Caretta, A., van Loosdrecht, P. H. M. & Palstra, T. T. M. (2012). *Chem. Mater.* **24**, 133–139.

[bb19] Sheldrick, G. M. (2008). *Acta Cryst.* A**64**, 112–122. 10.1107/S0108767307043930 18156677

[bb20] Sheldrick, G. M. (2015). *Acta Cryst.* C**71**, 3–8.

[bb21] Zhang, S. Y., Chen, W. Q., Hu, B., Chen, Y. M., Li, W. & Li, Y. (2012). *Inorg. Chem. Commun.* **16**, 74–77.

